# MicroRNA miR-106a-5p targets forkhead box transcription factor FOXC1 to suppress the cell proliferation, migration, and invasion of ectopic endometrial stromal cells via the PI3K/Akt/mTOR signaling pathway

**DOI:** 10.1080/21655979.2021.1933679

**Published:** 2021-06-04

**Authors:** Xinyue Zhou, Zhenyu Chen, Lipeng Pei, Jingli Sun

**Affiliations:** Department of Obstetrics and Gynecology, The General Hospital of Northern Theater Command, Shenyang, People’s Republic of China

**Keywords:** miR-106a-5p, FOXC1, endometriosis, PI3K, Akt, mTOR

## Abstract

Emerging evidence has exhibited an obvious decreased expression of miR-106a-5p in the ectopic endometrial tissue of endometriosis (EMS) patients. Thus far, the pathophysiological function of miR-106a-5p in EMS is unknown. A previous study showed an increased FOXC1 expression in the ectopic endometrial tissue of patients with EMS. Moreover, we found that there was a binding site of miR-106a-5p on the 3ʹUTR of FOXC1 through bioinformatics predictions. Hence, we speculated that miR-106a-5p might affect the development of EMS via targeting FOXC1. We first showed a decreased level of miR-106a-5p and an increased level of FOXC1 mRNA in ectopic endometrial tissues compared with normal tissues. Functionally, we transfected ectopic endometrial stromal cells (ESCs) with miR-106a-5p mimics or NC mimics and indicated an inhibitory role of miR-106a-5p on ESC proliferation, invasion, and migration. Mechanistically, FOXC1 was found to be a target gene of miR-106a-5p. To confirm whether miR-106a-5p exerted an inhibitory activity in ESCs via targeting FOXC1, miR-106a-5p mimic was co-transfected into ESCs with the FOXC1-plasmid or vector. We found that FOXC1 overexpression evidently reversed the results caused by a miR-106a-5p mimic in ESCs. Additionally, our results demonstrated that miR-106a-5p mimic inhibited the expression of p-Akt and p-PI3K. Collectively, these results revealed that miR-106a-5p inhibited the proliferative, migratory, and invasive ability of ESCs via directly binding to FOXC1, likely through the suppression of the PI3K and its downstream signaling pathway, which offered a potential and novel therapeutic strategy for EMS treatment.

## Graphical abstract

**Figure:**
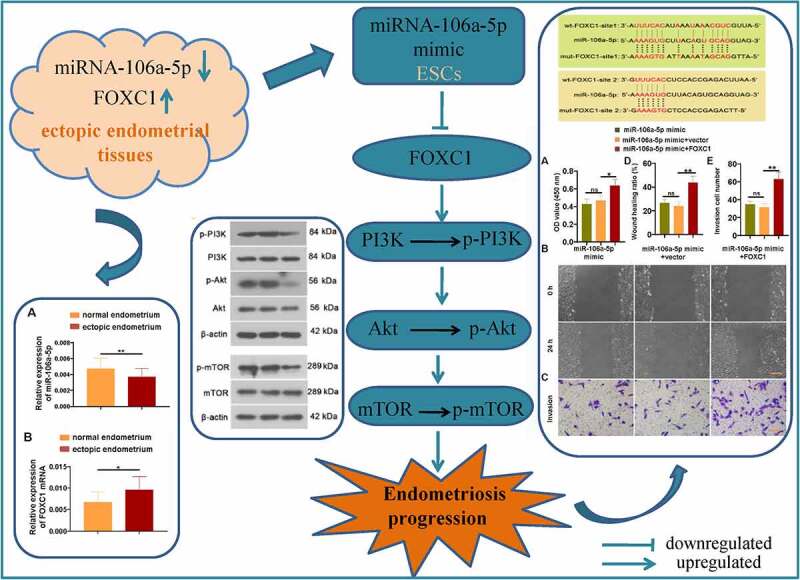

## 1. Introduction

Endometriosis (EMS) is a debilitating disease in which endometrial glands and stroma are planted outside the uterus, adversely affecting social, professional, and psychological functions [1]. It is estimated that the incidence of EMS reaches 10%-20% among women of reproductive age [2], rises as much as 30% among infertile patients, and increases to 45% among patients with chronic pelvic pain [3]. While the specific etiology of EMS remains a mystery, the currently recognized pathogenesis of EMS is the theory of menstrual blood reflux and endometrial implantation [4]. It is worth noting that the abnormality of the immune system, the causes of hereditary, and the unsuitable environment and lifestyle can also induce the occurrence of EMS besides blood reflux [5]. Recent research has implicated that the EMS diagnosis could be an early marker for ovarian endometrioid carcinoma diagnosis [6]. Studies have shown that in EMS patients, the proportion of retrograde cell apoptosis is greatly reduced, and the sensitivity of surviving ectopic cells to apoptosis is reduced, leading to excessive survival of the ectopic endometrium [7–9]. Moreover, the ectopic endometrium of EMS patients is more aggressive and easier to implant in the abdominal cavity [10]. So far, there is no pretty successful method to treat EMS. Clinical therapies are mainly divided into two types: one is pharmacological therapy designed to inhibit the growth of endometriotic implants, and the other is surgical therapy attempted to remove or destroy endometriotic implants [5]. However, these methods have their shortcomings and limitations [5]. Therefore, finding a more suitable treatment for EMS is the primary problem.

MicroRNAs (miRNAs) are a type of endogenous single-stranded RNA with a length of about 18–22 nucleotides, which can specifically recognize seed sequences to inhibit the translation of targeted mRNA or degrade mRNA and participate in a broad spectrum of cellular processes [11,12]. miR-106a-5p is usually used as a tumor inhibitor gene to regulate the abnormal proliferation, apoptosis, invasion, and migration of a variety of tumor cells [13–15]. A previous study has indicated that miR-106a-5p is decreased in the ectopic endometrial tissue of patients with EMS [16], but its role in the pathogenesis of EMS has not been reported yet. FOXC1 is a member of the FOX (forkhead box) transcription factor family, which participated in several biological processes [17]. The dysregulation of FOXC1 protein involves various physiological processes such as proliferation, differentiation, invasion, and metastasis [17–19]. Importantly, an increased FOXC1 expression was found in the ectopic endometrial tissue of patients with EMS [16]. Through bioinformatics predictions, we also found that there was a binding site of miR-106a-5p on the 3ʹUTR of FOXC1. Therefore, we speculated that miR-106a-5p might affect the development of EMS via targeting FOXC1.

In this study, we screened for the miR-106a-5p and FOXC1 expression in endometrial tissues from patients with EMS and identified that miR-106a-5p was at a declining level while FOXC1 was opposite. Then, we conducted a miR-106a-5p mimic model in ectopic endometrial stromal cells (ESCs). Functionally, the CCK-8, wound healing and transwell invasion assay were performed to explore the function of miR-106a-5p on ESC proliferation, migration and invasion. Moreover, previous studies have demonstrated that MMP2 and MMP9 are involved in cell migration, tumor growth and metastasis [20,21]. Hence, we further detected the expression of MMP2 and MMP9. Mechanistically, we found that miR-106a-5p targeted FOXC1 to exert the inhibitory effect in EMS development, likely via the PI3K/Akt/mTOR signaling pathway.

## 2. Materials and methods

### 2.1. Tissue samples

Ectopic endometrium (EC) samples were obtained from patients (n = 19, aged 23–48 years) with EMS. As controlled, normal endometrium (NE) samples were obtained from patients without EMS (*n* = 4, aged 33–48 years). In our study, there were 3 cases with early-stage EMS (stage II), 16 cases with advanced stage EMS (stage III or IV), and 4 cases without EMS. Moreover, there are 16 cases with dysmenorrheal and 7 cases without dysmenorrheal. None of the subjects had received hormones drugs for the 6 months before surgery. The level of miR-106a-5p and FOXC1 in collected EC or NE samples was first detected. Another collected EC sample was used to isolate ESCs for subsequent experiments. These endometrial specimens were approved by the Ethics Committee of the General Hospital of Northern Theater Command.

### 2.2. Cell culture

ESCs derived from EC tissues of women with EMS were isolated according to the method described previously [22,23]. Briefly, the EC tissues were cut into 0.5–1 mm^3^ pieces and digested in 5 mL DMEM/F12 (PM150312, Procell, China) supplemented with 0.1% collagenase (2275MG100, BioFroxx, German) at 37°C for 1 h. Subsequently, tissue residue was filtered through a 100-mesh screen and centrifuged at 800 g at 4°C for 5 min. Then the supernatant was discarded and ESCs were re-suspended, filtered through a 200-mesh screen, and centrifuged at 1000 g at 4°C for 10 min. After that, ESCs were collected and suspended in DMEM/F12 containing 10% (v/v) fetal bovine serum (FBS). The purity of the isolated ESCs was determined by an Immunofluorescent staining assay using antibodies for Cytokeratin (NBP2-29429, Novus, China) and Vimentin (16396-1-AP, Proteintech, China).

### 2.3. Cell transfection

Cell transfection was performed as described previously [24,25]. Briefly, ESCs were seeded in 12-well plates at 70% confluency and incubated at 37°C overnight. Subsequently, ESCs were transfected with miR-106a-5p mimic (JTS scientific, Wuhan, China), NC mimic, miR-106a-5p inhibitor, NC inhibitor, mutant FOXC1 3′-UTR, wild-type FOXC1 3′-UTR using Lipofectamine 3000 (Invitrogen) for 48 h.

### 2.4. Real-time quantitative PCR (RT-qPCR)

RT-qPCR was performed as described before [24]. Briefly, total RNA or miRNA from cultured ESCs and tissues was extracted by TRIpure (RP1001, BioTeke, China). Then, total miRNA was reversed transcribed using the miRNA First Strand cDNA Synthesis kit (#B532451, Sangon, Shanghai). Total RNA was converted into first-strand cDNA using SuperScript M-MLV reverse transcriptase (PR6502, BioTeke) with Exicycler^TM^ 96 Real-Time Quantitative Thermal Block (BIONEER, Korean). RT-qPCR was used to examine miR-106a-5p and FOXC1 expression using an SYBR Green Master Mix (Solarbio, Beijing). β-actin was served as an internal control. The relative expression of miR-106a-5p and FOXC1 was calculated in a 2^−∆∆CT^ manner. The primer sequences were exhibited as followed: hsa-miR-106a-5p, forward: 5ʹ- AAAAGTGCTTACAGTGCAGGTAG −3ʹ; FOXC1, forward: 5ʹ- ACAGCATCCGCCACAACCTC −3ʹ, reverse: 5ʹ- TGTCCTTCACCGCGTCCTTC −3ʹ; β-actin, forward: 5′- CACTGTGCCCATCTACGAGG-3′, reverse: 5′- TAATGTCACGCACGATTTCC-3′.

### 2.5. Western blot

Western blot assay was performed as described before [26]. Briefly, total proteins of ESCs were extracted with RIPA (P0013, Beyotime, China) containing PMSF (ST506, Beyotime). After the measurement of protein concentration by the BCA kit (P0011, Beyotime), proteins were SDS-PAGE electrophoresed, transferred to PVDF membranes (IPVH00010, Millipore, USA), incubated for 1 h with 5% skim milk, and incubated at 4°C overnight with following antibodies: MMP-9 (1: 500, 10375-2-AP, Proteintech, China), FOXC1 (1: 500, A2924, ABclonal, China), PI3K (1: 1000, AF6241, Affinity, China), p-PI3K (1: 1000, AF3242, Affinity), p-Akt (1: 1000, AF6261, Affinity), Akt (1: 1000, AF0016, Affinity). After 4 times washing, the membrane was immune-blotted with secondary antibody for 40 min at 37℃. Protein expression was normalized to β-actin (1: 2000, 60008-1-Ig, Proteintech).

### 2.6. Cell counting kit-8 (CCK-8) assay

ESC cell proliferation was measured by CCK-8 assay [22]. The treated ESCs were inoculated in a 96‐well plate at a density of 3 × 10^3^cells/well and further cultured for 24 h for complete cell adherence. CCK‐8 kit (96992, Sigma, USA) was employed to detect cell viability following the users’ manual. At last, the absorbance value was measured at 450 nm. The OD value was detected at 0, 24, 48, 72, and 96 h.

### 2.7. Wound healing assay

ESC cell proliferation was measured by wound healing assay [22,23]. ESCs were inoculated in a 6-well plate and transfected with plasmids (as the method described in *Cell transfection* above). When reached 95–100% confluence, ESCs were cultured in a serum-free medium and then incubated with 1 μg/mL mitomycin C (M0503, Sigma) for 1 h. Subsequently, ESCs were scratched with a 200-μL pipette tip across the center of the well and cultured in the medium with no serum for another 24 h. ESCs were visualized at 0 h and 24 h under a microscope.

### 2.8. Transwell invasion assay

Transwell invasion assays were used to examine the capacity of ESCs to invade [23,26]. Briefly, the inserts (3422, Corning, USA) coated with 40 μL the Matrigel matrix (356234, Corning) were placed in a 24-well plate and incubated at 37°C for 2 h. After transfection for 48 h, ESCs were collected and cultured into the upper Transwell chamber and invaded toward the lower chamber with 800 μL of mediumcontaining10% FBS for 24 h. Subsequently, the ESCs were photographed and counted.

### 2.9. Immunofluorescence staining

Cytokeratin and Vimentin are regarded as the markers of ESCs [27]. ESCs were fixed with 4% paraformaldehyde and permeabilized with 0.1% Triton X-100 (ST795, Beyotime). Then, ESCs were blocked at room temperature (RT) for 15 min with goat serum (SL038, Solarbio). Subsequently, ESCs were incubated at 4°C overnight with antibodies: Cytokeratin (1: 200) and Vimentin (1: 200) followed by incubation with secondary antibody at RT for 1 h. DAPI (D106471, Aladdin, China) was used to stain cell nuclei.

### 2.10. Luciferase reporter assay

Luciferase reporter assay was performed as shown before [23,28]. The FOXC1 sequence with the miR-106a-5p binding site was cloned into the pmirGLO vector (Promega, USA) to construct the binding site wild-type (wt-FOXC1-site) and mutant-type (mut-FOXC1-site). Then, these plasmids were co-transfected into ESCs with miR-106a-5p mimics or NC mimics according to the instruction of the luciferase assay kit (E1910, Promega). After cultured for 48 h, the luciferase activity was detected.

### 2.11. Statistical analysis

The data were analyzed with Graphpad Prism 8.0 (San Diego, CA, USA). All data were presented as mean ± standard deviation (SD). The student’s t-test was used to analyze the differences between the two groups. One-way ANOVA was applied when more than two groups. Two-way ANOVA was applied to analyze cell proliferation. P < 0.05 was identified significant difference.

## 3. Results

In this study, we conducted a miR-106a-5p mimic model in ESCs and performed the CCK-8, wound healing, transwell invasion assay as well as MMP2 and MMP9 detection to explore the function of miR-106a-5p on ESC proliferation, migration, and invasion. Moreover, we found that miR-106a-5p targeted FOXC1 to exert the inhibitory effect in EMS development, likely via the PI3K/Akt/mTOR signaling pathway.

### 3.1. The decreased level of miR-106a-5p and increased expression of FOXC1 in EMS

**Figure 1.**
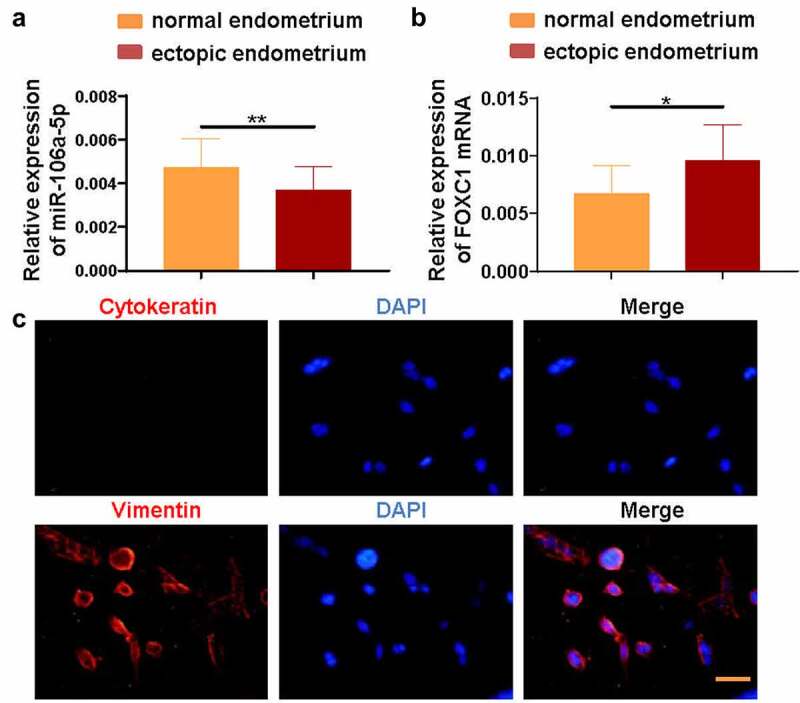
**miR-106a-5p was decreased and FOXC1 was increased in endometrial tissues**. (a) The RT-qPCR assay demonstrated a decreased level of miR-106a-5p in ectopic endometrial specimens compared with normal endometrial specimens. Student’s t-test, **p < 0.01. (b) FOXC1 mRNA level was increased in ectopic endometrial specimens compared with normal endometrial specimens. Student’s t-test, *p < 0.05. (c) An immunofluorescence assay was conducted to detect the expression of Vimentin and Cytokeratin in ectopic endometrial stromal cells (ESCs). DAPI was used to stain the nucleus (blue). Scale bar: 50 μm

To evaluate the expression level of miR-106a-5p and FOXC1 in EMS, RT-qPCR was performed to evaluate the level of miR-106a-5p and FOXC1 in both EC and NE samples. Our results exhibited a significantly decreased expression level of miR-106a-5p in EC tissues compared with NE tissues (P < 0.01; Figure 1(a)). By contrast, the expression of FOXC1 was evidently increased in the EC group compared with the NE group (P < 0.05; Figure 1(b)). To perform subsequent experiments, ESCs were isolated from EC samples. Hence, we detected Cytokeratin and Vimentin expression via immunofluorescent staining, which determined the purity of the isolated ESCs (Figure 1(c)).

### 3.2. miR-106a-5p inhibited ESC proliferation, migration, and invasion

**Figure 2.**
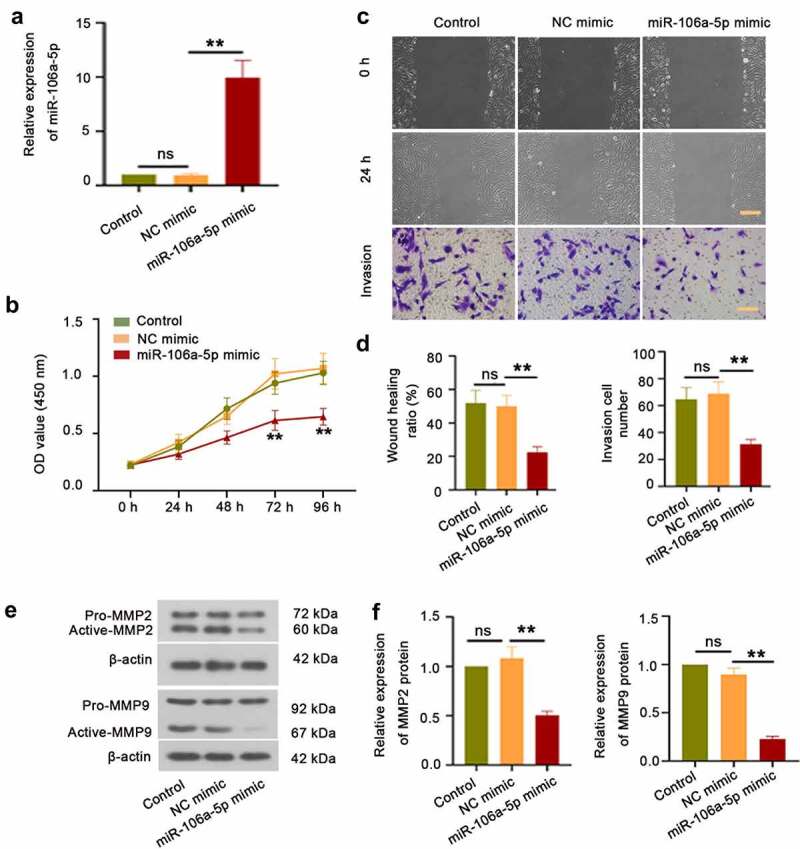
**miR-106a-5p repressed ESC proliferation, invasion, and migration**. (a) The expression of miR-106a-5p in ESCs with miR-106a-5p mimic transfected. One-way ANOVA. **p < 0.01, ns: not-significant. (b) The CCK-8 assay was performed to examine the cell viability of ESCs transfected with miR-106a-5p mimic. Two-way ANOVA. **p < 0.01, ns: not-significant. (c, d) A wound-healing assay and the transwell invasion assay were carried out to measure the cell migration and invasion of ESCs transfected with miR-106a-5p mimic. Scale bar in migration: 200 μm. Scale bar in invasion: 100 μm. (e) The western blot assay was used to detect MMP2 and MMP9 expression in ESC with miR-106a-5p mimic transfected. β-actin was conducted as a loading control. (f) The quantification of MMP2 and MMP9 protein. One-way ANOVA. **p < 0.01, ns: not-significant

To evaluate the effect of miR-106a-5p on ESCs, we transfected ESCs with miR-106a-5p mimic or NC mimic. RT-qPCR results demonstrated that miR-106a-5p mimic was successfully transfected into ESCs (Figure 2(a)). The CCK-8 assay exhibited an obvious inhibition of cell proliferation in ESCs transfected with miR-106a-5p mimic at 72 h and 96 h compared with NC mimic (Figure 2(b), p < 0.01). Moreover, a wound-healing assay of ESCs was carried out to observe the effects of miR-106a-5p in ESC migration. The results showed that ESCs transfected with miR-106a-5p mimic suppressed the migration and invasiveness of ESCs (Figure 2(c,d), P < 0.01). Meanwhile, we detected the expression of MMP2 and MMP9, which are involved in cell migration and invasion [20,21]. Western blot results showed a decreased level of MMP2 and MMP9 with activated form in ESCs transfected with miR-106a-5p mimic than that in ESCs transfected with NC mimic (Figure 2(e,f)). Additionally, ESCs were also transfected with miR-106a-5p inhibitor or NC inhibitor. The results were opposite to the miR-106a-5p mimic. As shown in Figure S1A-D, miR-106a-5p inhibitor exerted a promoting effect on ESC viability, migration, and invasion. Collectively, our data indicated that miR-106a-5p inhibited ESC proliferation, invasion, and migration.

### 3.3. miR-106a-5p targeted 3ʹUTR of FOXC1 and repressed the PI3K/Akt/mTOR signaling pathway

**Figure 3.**
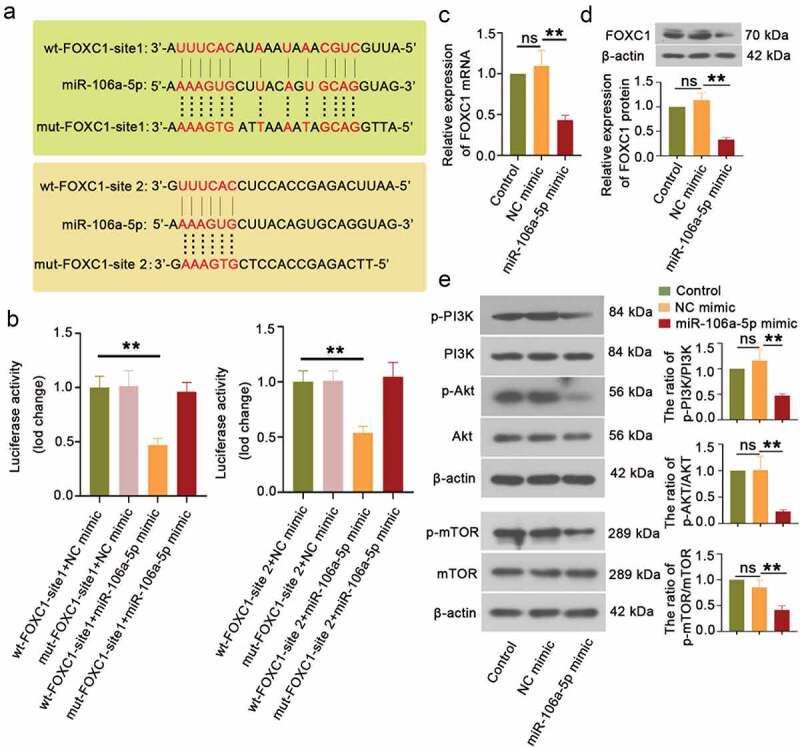
**miR-106a-5p downregulated FOXC1 expression via targeting 3ʹUTR of FOXC1**. (a) The sequence alignment of FOXC1 binding to miR-106a-5p. (b) Luciferase reporter assay was performed to evaluate the binding activity between FOXC1 and miR-106a-5p. One-way ANOVA. **p < 0.01. (c) The FOXC1 expression in ESCs with miR-106a-5p mimic transfected was examined using RT-qPCR. One-way ANOVA. **p < 0.01, ns: not-significant. (d, e) The detection and quantification of FOXC1, the p-PI3K, p-AKT, and p-mTOR in ESCs with miR-106a-5p mimic transfected using western blot assay. β-actin was conducted as a loading control. One-way ANOVA. **p < 0.01, ns: not-significant

To explore the molecular mechanism of miR-106a-5p in regulating the proliferative, migratory, and invasive ability of ESCs, the predicted results from ENCORI showed that miR-106a-5p might target 3ʹUTR of FOXC1. As shown in Figure 3(a), the sequence alignment of FOXC1 bound to miR-106a-5p. The site types of wt-FOXC1-site 1 and wt-FOXC1-site 2 were 7mer-A1 and 6mer respectively. Compared to other groups, a significantly decreased luciferase activity was observed in ESCs with wt-FOXC1 and miR-106a-5p mimic co-transfected (Figure 3(b), p < 0.01). Furthermore, RT-qPCR results demonstrated that miR-106a-5p mimic reduced the level of FOXC1 mRNA in ESCs (Figure 3(c)). Western blot results exhibited the same trend of FOXC1 expression as RT-qPCR results (Figure 3(d)). In addition, we also found the miR-106a-5p mimic inhibited the expression of the phosphorylation of PI3K, Akt, and mTOR (Figure 3(e)). Taken together, we showed that miR-106a-5p directly bound to 3ʹUTR of FOXC1 and suppressed the PI3K/Akt/mTOR signaling pathway.

### 3.4. miR-106a-5p suppressed ESC proliferation, invasion, and migration by reducing FOXC1 expression

**Figure 4.**
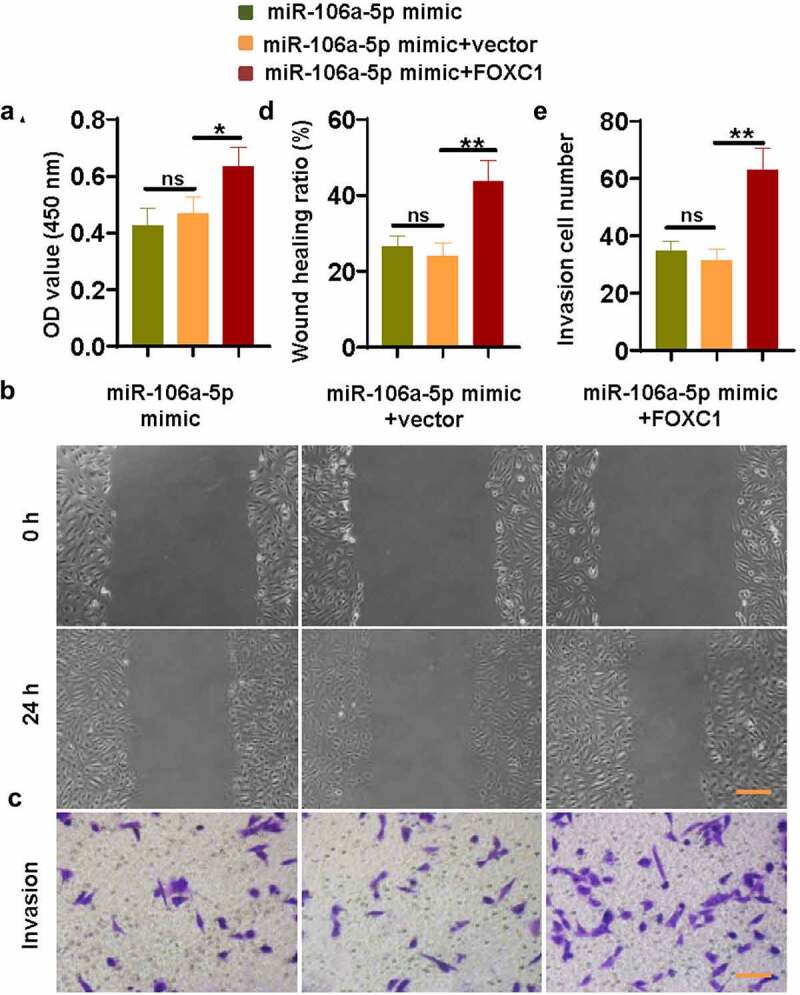
**miR-106a-5p inhibited ESC proliferation, invasion, and migration via reducing FOXC1 expression**. (a) The CCK-8 assay revealed that FOXC1 promoted the proliferation of ESCs which were suppressed by a miR-106a-5p mimic. (b) The down-regulated migration activity of ESCs caused by the miR-106a-5p mimic was activated by FOXC1. Scale bar: 200 μm. (c) The declined invasion activity of ESCs caused by the miR-106a-5p mimic was promoted by FOXC1. Scale bar: 100 μm. (d-e) The quantitative analysis of wound healing ratio and invasion cell number. One-way ANOVA. *p < 0.05, **p < 0.01, ns: not-significant

To explore whether miR-106a-5p suppressed ESC proliferation, invasion, and migration via targeting FOXC1, ESCs were co-transfected with miR-106a-5p mimic and FOXC1-plasmid or vector. Then the ability of ESCs to proliferate, migrate and invade was detected respectively. Our results suggested that FOXC1 reversed the suppression of ESC proliferation, migration, and invasion activities caused by miR-106a-5p mimic (Figure 4(a–e)). These data revealed an inhibitory effect of miR-106a-5p in ESC proliferation, invasion, and migration through reducing the expression of FOXC1.

## 4. Discussion

EMS is associated with pain and infertility, which manifests during reproductive years [29]. Although pain can be alleviated through pharmacological suppression of ovulation and menstruation or surgery, lesions are not eradicated and the benefit is often temporary [29]. Hence, it is necessary to explore a novel way to treat EMS. Herein, we indicated that miR-106a-5p was decreased in EC samples from patients with EMS and miR-106a-5p mimic in ESCs might suppress ESC proliferation, invasion, and migration through reducing the expression of FOXC1, likely via the PI3K and its downstream signaling pathway.

Multiple miRNAs (such as miR-451, miR-125b, miR-145, miR-194, and miR-199a) are involved in EMS development and regulation [16,30]. However, no study has yet reported the function and mechanism of miR-106a-5p in EMS. In the present work, we demonstrated that decreased miR-106a-5p levels were observed in the EU when compared to the NE tissues, which provided clinical insight into the contribution of miR-106a-5p to the pathophysiological regulation of EMS. The more clinical samples, the more representative they are, including the NE samples. There is a limitation that we have tried our best but the sample size is still not satisfactory. But it is worth noting that a previous study detected the miR-106a-5p level in 30 paired ectopic endometrium (EC) (endometriotic lesions) and the eutopic endometrium (EU) tissues, and the results showed that miR-106a-5p is down-regulated in EC [16]. Combined with our data, we have reasons to believe that the reduced miR-106a-5p level is involved in the pathogenesis of endometriosis.

In the subsequent study, ESC proliferation, invasion, and migration were inhibited by a miR-106a-5p mimic. Additionally, studies have reported that matrix metalloproteinases are a miRNA target related to EMS, and their expression is increased in the EU, mainly by degrading the extracellular matrix, helping tissue remodeling and promoting intrauterine membrane migration and invasion [30,31]. Jana S et al. found that Curcumin could delay the development of EMS by inhibiting MMP2 activity [32]. Paul. S et al. suggested that Melatonin could protect against EMS via downregulation of MMP9 [33]. Consistent with these results, we also found decreased MMP2 and MMP9 after treatment with miR-106a-5p mimic, which further confirmed the inhibitory effect of miR-106a-5p in ESC proliferation, migration, and invasion. Interestingly, several studies have reported the inhibitory effect of miR-106a-5p in cell proliferation, invasion, and migration [13,14], which further confirmed our results.

Furthermore, we defined a new mechanism in this study whereby miR-106a-5p participated in regulating FOXC1 expression in EMS. Previous research has observed an upregulated expression of FOXC1 in the ectopic endometrial tissue of patients with EMS [16]. Our data exhibited that the change of miR-106a-5p in ESCs resulted in an alteration in the amount of FOXC1 protein. Furthermore, our experimental evidence from in vitro studies strongly suggested that FOXC1 could be a functional target of miR-106a-5p and might mediate its regulation role in ESC proliferation, migration, and invasion. Consistent with our results, there is evidence that FOXC1 overexpression contributes to an increased capacity for cancer cell survival and proliferation [17]. Another research has indicated FOXC1 can promote colorectal cancer cell proliferation by enhancing the Warburg effect [34]. Zuo et al. has demonstrated the effect of FOXC1 in mediating the proliferation, migration, cell cycle, and epithelial-mesenchymal transition of basal-like breast cancer cells [35]. Some compelling evidence has pointed that ectopic overexpression of FOXC1 in human basal-like breast cancer cells can contribute to increased tumor cell proliferation [36].

Interestingly, several studies have indicated that FOXC1 regulates cell proliferation, invasion, and migration via activating the PI3K and its downstream Akt signaling pathway [37–39]. The PI3K/Akt signaling has been involved in the development of EMS [40–44]. Recent research has indicated that the inhibition of the PI3K/Akt/mTOR pathway might be a therapy for EMS treatment [45]. In the present work, we also indicated that miR-106a-5p targeted FOXC1 to inhibit cell proliferation, invasion, and migration by inhibiting the PI3K and its downstream Akt pathway in our study. Similarly, research has reported that miR-106a-5p can regulate C2C12 myogenesis via repressing the PI3K/Akt pathway [46]. It is noteworthy that several reports have demonstrated that the PI3K/Akt signaling participated in the regulation of cell proliferation, invasion, and migration in different cancer cells [47–49], which further provides possible pieces of evidence for the inhibitory effect of miR-106a-5p in EMS via the PI3K/Akt/mTOR signaling pathway. In addition, the main disadvantage of our study is that there are too few cases in the normal group in the clinical sample.

## 5. Conclusion

Taken together, it is the first time for our study to provide a new finding that miR-106a-5p suppressed the proliferative, invasive, and migratory ability of ESCs *in vitro*, and up-regulation of miR-106a-5p can be used for treating EMS in pathological settings.

## Supplementary Material

Supplemental Material

## Data Availability

The data used to support the findings in this study are available from the corresponding author upon request

## Research highlights

- miR-106a-5p mimic repressed ESC proliferation, invasion, and migration
- miR-106a-5p bound to FOXC1
- miR-106a-5p suppressed the PI3K and its downstream signaling pathway
- miR-106a-5p inhibited EMS progression via targeting FOXC1
