# Supplementary material for: MicroRNA miR-106a-5p targets forkhead box transcription factor FOXC1 to suppress the cell proliferation, migration, and invasion of ectopic endometrial stromal cells via the PI3K/Akt/mTOR signaling pathway
Source: Bioengineered. 2021 Jun 4;12(1):2203–13. doi: 10.1080/21655979.2021.1933679 (PMC8806537; doi:10.1080/21655979.2021.1933679)
Supplement: Supplemental Material [file KBIE_A_1933679_SM0133.zip › Supplementary/Supplementary figure 1.docx]

**Supplementary figure 1. miR-106a-5p inhibitor increased viability, invasion and migration activity of ESC.** (A) The CCK-8 assay was performed to examine the cell viability of ESCs transfected with miR-106a-5p inhibitor. One-way ANOVA. *p < 0.05, ns: not-significant. (B) A wound-healing assay was carried out to measurethe cell migration of ESCs transfected with miR-106a-5p inhibitor. Scale bar: 200 μm. The transwell invasion assay was used to assess ESC invasion. Scale bar: 100 μm. (C)The wound-healing score. One-way ANOVA. *p < 0.05, ns: not-significant. (D) The invasion cell number of transwell invasion assay. One-way ANOVA. **p < 0.01, ns: not-significant.
